# Evidence of Spatial Synchrony in the Spread of an Invasive Forest Pest

**DOI:** 10.1111/ele.70140

**Published:** 2025-05-30

**Authors:** Clare A. Rodenberg, Jonathan A. Walter, Kyle J. Haynes

**Affiliations:** ^1^ Department of Environmental Sciences University of Virginia Charlottesville Virginia USA; ^2^ Center for Watershed Sciences University of California Davis California USA; ^3^ Blandy Experimental Farm University of Virginia Boyce Virginia USA

**Keywords:** invasive spread, population synchrony, range expansion, spongy moth, teleconnection

## Abstract

Because population growth is a key component of range expansion, spatial synchrony in population growth along a species' range edge may lead to spatial synchrony in range expansion. However, demographic stochasticity in low‐density range‐edge populations and stochastic long‐distance dispersal may disrupt the synchronisation of range expansion. Here, we investigate whether rates of spread by an invasive species, the spongy moth and exhibit spatial synchrony. We also evaluate if climatic oscillations at multi‐annual timescales arising from teleconnections synchronise spread at similar timescales. We applied extensions of wavelet analysis to spatiotemporal data on climate variables and range‐edge abundances during 1990–2020. Synchrony in spread occurred throughout the entire study area, but only in the northernmost and southernmost ecoregions was synchrony in spread explained by multi‐annual climate oscillations linked to teleconnection patterns. We demonstrate spatial synchrony in invasive spread and find an opportunity to predict the timing of pulses of invasive spread at regional scales.

## Graphical abstract

Although synchrony in population growth rates and abundance is ubiquitous across many taxa, this study demonstrates a new manifestation of this phenomenon, that of spatial synchrony in range expansion. Just as climatic drivers can produce synchrony in population growth or abundance, we show that synchronised fluctuations in seasonal climate conditions, such as those created by teleconnection patterns, can synchronise spread rates of the spongy moth, a major forest pest in North America. If prevalent, this phenomenon could prove critical to management efforts aimed at either controlling the spread of pests and disease vectors or facilitating the movement of rare species as the climate changes.

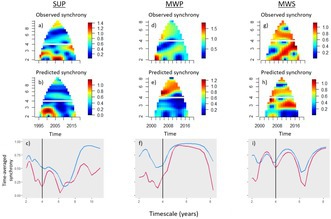

## 1 Introduction

Population spatial synchrony is a pervasive feature of population dynamics whereby fluctuations in spatially disjunct populations' growth or abundances are coherent through time (Bjørnstad et al. 1999; Liebhold et al. 2004; Reuman et al. 2023). This phenomenon is thought to result from regional correlations in environmental conditions (e.g., weather) that impact population growth (known as ‘Moran effects’; Moran 1953), dispersal and trophic interactions. Population synchrony has important ecological implications including increasing extinction risk for metapopulations (Abbott 2011; Heino et al. 1997), and the synchronisation of pest‐species population cycles across wide areas resulting in outbreaks that are more destructive at regional scales (de Valpine et al. 2010; Liebhold et al. 2012). Just as regionalized outbreaks of pests are more concerning from a management perspective than localised ones (Liebhold et al. 2012), a management agency's ability to slow invasion rates or mitigate negative impacts of the invasion would likely be low during a regionally synchronous pulse in spread. Yet, we know very little about the pervasiveness of spatial synchrony in rates of range expansion or contraction or its drivers.

Population growth is a key component of range expansion (Skellam 1951), suggesting that spatial synchrony in population growth along a species' range edge may lead to spatial synchrony in range expansion. However, other factors may work against the synchronisation of range expansion. For example, the greater impacts of demographic stochasticity on low‐density populations (Lande 1993; May 1974) could disrupt regional synchronisation of population growth and, in turn, range expansion, because range‐edge populations tend to be low‐density (Shigesada and Kawasaki 2002; Walter et al. 2020). In addition, rates of range expansion are strongly influenced by stochastic long‐distance dispersal events (Caswell et al. 2003; Shigesada and Kawasaki 2002), which could desynchronize range expansion at a regional scale.

Spectral approaches to studying synchrony have shown that population and community synchrony often exhibit timescale structure, with fluctuations that are synchronous on some timescales (i.e., periods, the inverse of frequencies) but asynchronous on others (Anderson et al. 2019; Defriez et al. 2016; Vasseur and Gaedke 2007). The timescale structure in population synchrony has proven useful for drawing conclusions about its drivers (Anderson et al. 2019; Haynes et al. 2019; Sheppard et al. 2016). For example, populations of some insect species exhibit synchrony at the same multi‐year timescales as weather variables (Haynes et al. 2019; Walter et al. 2017). Sheppard et al. (2016) revealed a strong link between a climate teleconnection, the North Atlantic Oscillation, and spatial synchrony in the timing of aphid first flights at long (> 4 years) timescales. Given the important role of population growth in spread, timescale‐specific synchrony in climate could spatially synchronise rates of range shifts over similar timescales, but this has not been investigated.

One reason why spatial synchrony of invasive spread is understudied is that spatiotemporal data on population abundances, especially for low‐density populations, are notoriously difficult to gather (Grayson and Johnson 2018). Low‐density populations along the outer range limits of a species provide a large fraction of the propagules for range expansion events (Lockwood et al. 2013). Therefore, population dynamics along the range edge strongly influence whether the species' range will expand, stabilise, or contract (Sexton et al. 2009).

The invasive spongy moth ( *Lymantria dispar* ) in North America is an ideal organism for studying spatial synchrony in spread. This damaging forest pest has been extensively monitored by government agencies since its original introduction to North America from Europe in the late 1860s (Tobin et al. 2012; Wu et al. 2015). Annual monitoring conducted by the Slow the Spread (STS) Programme tracks spongy moth densities along the species' range edge and has facilitated research on topics including range expansion and Allee effects (Coleman and Liebhold 2023; Grayson and Johnson 2018). Range expansion by the spongy moth displays periodic pulses, which in theory may be caused by Allee effects in newly colonised populations coupled with stratified diffusion (Johnson et al. 2006). Patterns of synchrony in spread, however, remain understudied in this and other organisms.

Here, we investigated spatial synchrony in rates of invasive spread by the spongy moth and potential drivers. Specifically, we evaluated the hypothesis that synchrony in the fluctuations of climate at multi‐annual timescales leads to spatial synchrony in rates of spread at similar timescales. To achieve this, we examined the spatial synchrony of rates of spread and climatic conditions within and across five previously defined (Omernik 1987; Omernik and Griffith 2014) ecoregions (i.e., areas with similar climatic and biotic conditions) from 1990 to 2020 using extensive spatiotemporal abundance and weather data. We focused on four climate variables that prior work suggests have impacts on spongy moth survival or reproduction (Logan et al. 1991; Siegert et al. 2008; Smitley et al. 1995; Streifel et al. 2019; Summers 1922; Thompson et al. 2017)—mean temperature and precipitation during the larval period (spring), and minimum temperature and snow depth during the egg life stage (winter). We anticipated that these variables would serve as drivers of synchronised spread of the spongy moth, depending on ecoregion and life stage. Because climate oscillations resulting from drivers of teleconnection patterns create multi‐annual, periodic fluctuations in weather across broad areas (Nigam and Baxter 2015), we expected that teleconnection sources (e.g., El Niño‐Southern Oscillation) would explain synchrony in the climate variables used for this study.

## 2 Material and Methods

### 2.1 Study System

**FIGURE 1:**
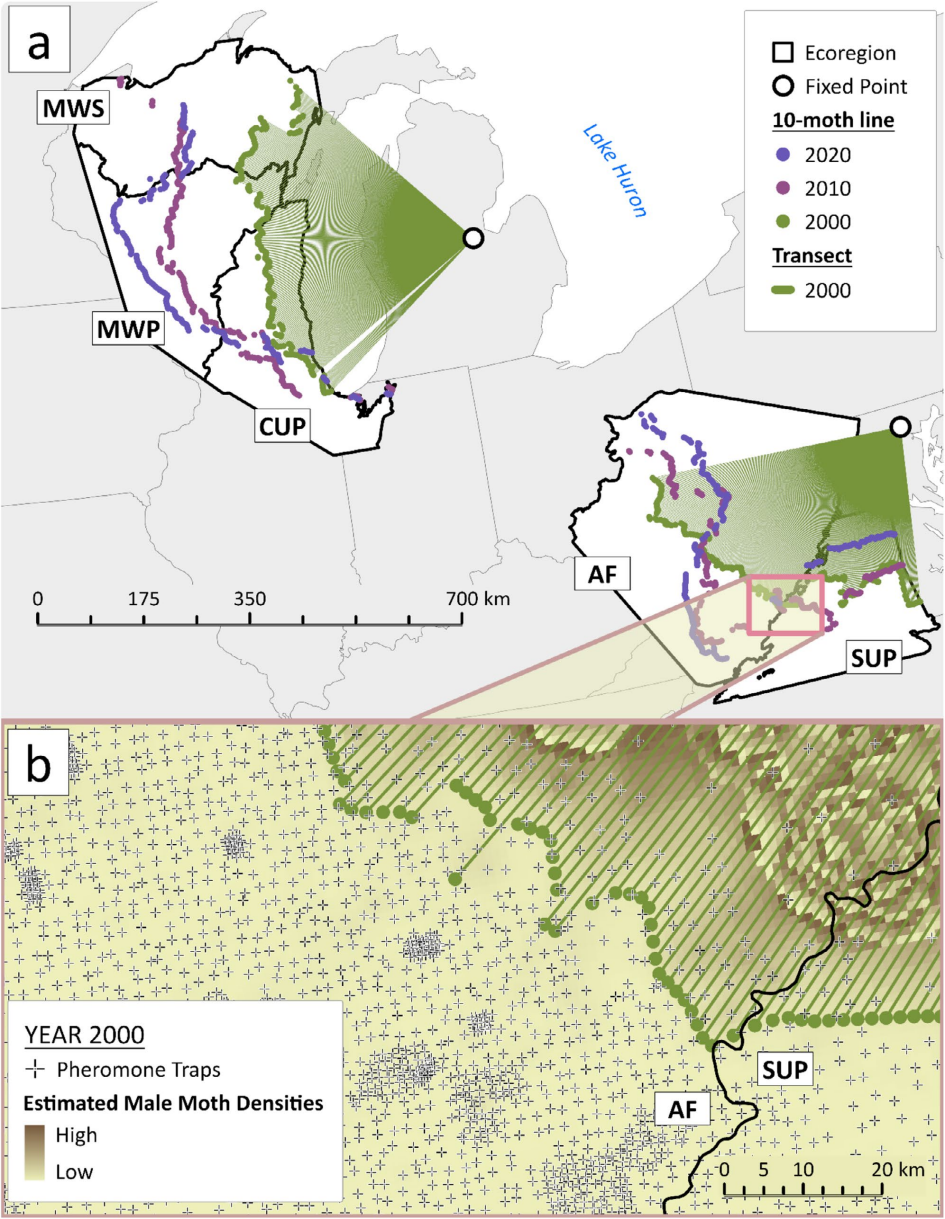
Sample of the raw spongy moth data used in this study. Panel a—Map of the study area delineated with EPA Level II Ecoregions (Omernik 1987; Omernik and Griffith 2014)—Mixed Wood Shield (MWS); Mixed Wood Plains (MWP); Central USA Plains (CUP); Appalachian Forest (AF) and Southeastern USA Plains (SUP). Green straight lines are evenly spaced (0.5°) transects radiating from the two fixed points used to create 10‐moth isoclines for the estimation of annual spread rates. The northern point is located at: 43.6° N, −84.2° W (Tobin et al. 2007) and southern point at: 39.4° N, −76.6° W (Tobin et al. 2014). Estimated 10‐moth isoclines for years 2000, 2010 and 2020 are displayed by a series of points that represent where each transect intersects the invasion front. Panel b—The underlying trap catch and interpolated data used to determine the location of a 10‐moth isocline. Hatch marks represent the location of pheromone‐baited traps deployed in year and used by the STS Program to catch male moths in 2020. The underlying smoothed, coloured surface ranging from light brown to dark brown, is the kriging interpolated surface of estimated moth densities.

During the winter, temperatures in the northernmost ecoregions of the spongy moth's invasive range in North America (Figure 1a) are often below the critical limit for egg survival (Streifel et al. 2019); however, snow may insulate eggs against lethal winter temperatures (Streifel et al. 2019; Summers 1922). In the warmest and southernmost ecoregions of the range, springtime temperatures can be supraoptimal for larval and pupal development (> 28°C; Logan et al. 1991), reducing larval survival (Thompson et al. 2017) and supraoptimal temperatures have been linked to range retraction (Tobin et al. 2014; but see Rodenberg 2023). Precipitation during the spring affects larval survival because infection of larvae by the host‐specific fungal pathogen, *Entomophaga maimaiga*, increases with environmental moisture (Siegert et al. 2008; Smitley et al. 1995). In all but the coldest ecoregions, temperatures are generally mild enough for the persistence of the fungus (Siegert et al. 2009).

Although rates of spongy moth spread are influenced by seasonal and annual weather conditions (Rodenberg 2023; Tobin et al. 2014), the spongy moth exhibits periodicity in rates of spread, with pulses in spread occurring at longer timescales, roughly every 4–6 years (Johnson et al. 2006; Walter et al. 2015). Within the North American range of the spongy moth, precipitation and temperature exhibit multi‐annual periodicity (Allstadt et al. 2015; Haynes et al. 2019) and can display synchrony over distances up to 2500 km (Koenig 2002; Koenig and Liebhold 2016). It is unknown whether timescale‐specific synchrony in climate similarly impacts the spatiotemporal dynamics of the spongy moth's invasive spread.

### 2.2 Study Area

Since 2000, the STS Programme has tracked spongy moth abundances along the invasion front by deploying up to 200,000 pheromone‐baited traps annually (Coleman and Liebhold 2023). Specifically, managers deploy traps across the ‘transition zone’, a ~170 km wide swath of land along the spongy moth's range edge, including both recently infested areas and places still unoccupied the pest (Sharov et al. 2002). To ensure the range edge is near the middle of the transition zone the STS programme moves the zone boundary each year as the spongy moth spreads. Inter‐trap distances near the range edge are approximately 2 km. This transition zone was the study area for this investigation. To assess how patterns of spatial synchrony in spread and climate varied across the spongy moth's invasive range, we divided the invasion front into five ecoregions—Mixed Wood Shield (MWS), Mixed Wood Plains (MWP), Central United States Plains (CUP), Appalachian Forest (AF) and Southern United States Plains (SUP) (Figure 1a)—based on the Environmental Protection Agency's Level II Ecoregions (Omernik 1987; Omernik and Griffith 2014).

### 2.3 Data Processing

To estimate annual rates of spread, we used STS trap catch data from 1990 to 2020. First, we converted the data on moth densities (male moths/trap) into a smooth surface of densities at a 1 × 1‐km resolution using a median indicator kriging interpolation method (Isaaks and Srivastava 1989). We excluded areas within 1.5 km of spongy moth population suppression treatments. Only a small portion of the dataset was removed because STS treats < 2% of the monitoring area each year (Tobin et al. 2012). The estimated smoothed surface of spongy moth abundances contains inherent biases, especially as pheromone trap efficiencies change with the environment (Elkinton and Cardé 1988; Tobin et al. 2011); however, analyses using spongy moth trapping data have repeatedly proven to be successful for studying this species' range dynamics (Grayson and Johnson 2018).

The location of the spongy moth's range edge each year was represented by a population isocline where densities were equal to 10 moths per trap (Figure 1b). This so‐called ‘10‐moth line’ is the most stable in space and time compared with isoclines of other densities used by the STS programme (1, 3, 30, 100 and 300 moths) (Sharov et al. 1995, 1997). To estimate annual rates of range expansion or contraction, we used the boundary displacement method (Figure S1), which estimates the displacement (distance) between population isoclines for successive years along evenly spaced (0.5°) transects (Sharov et al. 1995, 1997; Tobin et al. 2007). The transects radiated to the 10‐moth line from fixed locations, chosen to ensure that the majority of transects intersected the isocline at as close to 90° as possible (Tobin et al. 2007). The coordinates of the fixed points were: 43.6° N, −84.2° W for the MWS, MWP and CUP (Tobin et al. 2007); 39.4° N, −76.6° W for the AF and SUP (Tobin et al. 2014) (Figure 1a). With this sampling arrangement, we estimated the annual radial rate of spread for each ecoregion based on the distance between the intersections of each transect and consecutive 10‐moth lines (Figure S1). We carried out all processes for estimating spread rates using ArcGIS Pro 3.1 software.

We defined winter as December–February and estimated the date range of the larval life stage for a given year and cell based on outputs of a temperature‐driven phenology model for spongy moth (Gray et al. 2001; Régnière and Sharov 1997, 1998, 1999) using BioSIM software (Régnière 1996). We estimated the larval date range for each location where a transect intersected with the 10‐moth line. For each year, we then obtained mean temperature and precipitation data at a 4‐km resolution for each location's estimated larval period (Daly et al. 1994). These data were from the Parameter‐elevation Regressions on Independent Slopes Model (PRISM; Daly et al. 1994).

For the winter period, we extracted daily values for minimum temperature and snow depth from PRISM (Daly et al. 1994) and the National Snow and Ice Data Center (Broxton et al. 2019), respectively, also at a 4‐km resolution. Because the calendar year turns over during the middle of winter and spread rates are estimated from pheromone trap catches of adult males during the summer, we used the wintertime data from the winter that preceded summertime trap catch efforts. We then calculated mean values of the larval and wintertime climatic variables within a circular buffer centered at the point of intersection between each transect and the 10‐moth line (Figure S1). The diameter of a buffer equalled each ecoregion's mean annual displacement distance (km) of the 10‐moth line from 1990 to 2020.

To understand whether synchronous multi‐annual fluctuations in the climate variables can be attributed to teleconnection patterns, we calculated mean monthly values for the North Atlantic Oscillation (NAO), the multivariate El Niño‐Southern Oscillation Index (MEI), and Pacific Decadal Oscillation (PDO) for the months that corresponded to the larval period and wintertime (December–February) for each ecoregion. These teleconnections were chosen because they influence temperature, precipitation, and snow depth within the study area (Wei et al. 2021; Whan and Zwiers 2017; Yu et al. 2012). Because the date ranges of the larval period differ between ecoregions, for each ecoregion, we calculated the mean values for the climate oscillation indices over the months that contained the larval‐period date ranges that were predicted using BioSIM (Régnière 1996).

### 2.4 Statistical Analyses

We used wavelet‐based approaches including wavelet mean fields (WMFs) and spatial wavelet coherence (Sheppard et al. 2016, 2017) to assess the prevalence of spatial synchrony in invasive spread and whether multi‐annual fluctuations in the climate variables—mean temperature and precipitation during the larval period (spring), and minimum temperature and snow depth during the egg life stage (winter)—are drivers of spatial synchrony in spread rates. Prior to analysing these relationships, each annual time series of the climate variables and spread rates was normalised using a Box–Cox transformation, detrended linearly, demeaned, and standardised to a variance of one (Sheppard et al. 2016). Unlike most studies on spatial synchrony which use stationary population data, our data were dynamic since range movement was the focus. Therefore, to study spatial synchrony of spread rates, we used transects approximately perpendicular to the 10‐moth line (Figure 1a and Figure S1) as the spatial unit for analysis. Wavelet‐based approaches require relatively long, yet equal length time series. To meet these criteria, for each ecoregion we (a) excluded time series < 20 years in length (Table S1) and (b) ensured that time series were the same length by dropping years with fewer than 10 transects.

We used WMFs to assess patterns of spatial synchrony in spongy moth spread rates (Sheppard et al. 2016). WMFs quantify the strength of synchrony as a function of time and timescale by measuring whether oscillations in a set of time series have aligned phases and correlated magnitudes (strengths) through time, as a function of timescale (Sheppard et al. 2016). If the phases of population oscillations are aligned, this indicates phase synchrony (phase locking in other contexts) (Blasius et al. 1999; Rosenblum et al. 1996). Perfect synchrony occurs when both the phases and magnitudes of the population oscillations are synchronised. To test the statistical significance of phase synchrony in spread rates, we compared magnitudes of wavelet phasor mean fields (WPMFs) to the null hypothesis of random (i.e., unsynchronized) phases or no synchrony (Sheppard et al. 2013, 2016, 2017) using the significance threshold of *p* < 0.001 (Sheppard et al. 2019). No significance test currently exists for the WMF due to difficulties of establishing an appropriate null hypothesis, so the significance tests using the WPMF complement and corroborate patterns observed in the WMF. Interpretation of the results for both WMFs and WPMFs are similar, with values ranging from 0 to 1 and higher values represent greater synchrony or phase synchrony, respectively.

To evaluate whether the climate variables were drivers of synchronised fluctuations of spread, we examined spatial wavelet coherence between each climate variable and spread rates (Sheppard et al. 2016, 2017). This technique reveals whether pairs of variables have phase differences and magnitudes of oscillations that are consistent through time and across space, as a function of timescale. Coherence between two variables, such as weather and population growth, is a strong indication that the environmental variable drives synchrony in the biological variable, providing evidence of transmission of synchrony from the environment to the population (Sheppard et al. 2016).

Phase differences provide information on the temporal lag between oscillations of two variables. No lag is present when variables are in‐phase or synchronous with each other, whereas anti‐phase or asynchronous dynamics occur when there is a lagged relationship. Mean phase differences ($\bar{\theta }$) are reported in fractions of π and range from 0 (in‐phase) to ±1 (anti‐phase), with intermediate values representing fractional (e.g., ¼‐cycle) lagged relationships. Significance testing of coherence was based on surrogate datasets representing a null hypothesis of no coherence that were generated using a randomization procedure that retains the spatial and temporal autocorrelation present within the raw data (Sheppard et al. 2016, 2017). We compared spatial coherences from 2000 surrogate time series to spatial coherence values for the observed data for two separate timescale bands—short timescales as 2–4‐year period lengths, and long timescales as > 4‐year periods. These timescales were chosen because the 4‐year cutoff separates cycles that are negatively lag‐1 autocorrelated (short timescale) from those that are positively lag‐1 autocorrelated (long timescale) (Sheppard et al. 2016). Following Walter et al. (2020), significance was evaluated at the *p <* 0.1 threshold due to the conservative nature of the test, though most nominally significant relationships had *p* < 0.05.

After identifying the climate variables that were coherent with rates of spread, we applied the wavelet Moran theorem (Sheppard et al. 2016) to quantify the percentage of synchrony in spread that can be explained by synchronous, multi‐annual climatic fluctuations (Table 1). We then calculated ‘cross‐terms’, a diagnostic of an independence assumption of the wavelet Moran theorem. Large cross‐terms (> 10%) indicate the assumption is unmet; specifically, large cross‐terms mean that the unexplained synchrony in one location is correlated with the effect of the climate variable at other locations (Sheppard et al. 2016). Lastly, we tested for significant coherence between timescale‐specific synchrony in the climate variables that were synchronous with spread (Table 1) and climate indices representative of the teleconnections (Table 2). The significance test procedures used for both WPMFs and spatial coherence account for the number of transects in each ecoregion. To employ the wavelet methods, we used the ‘wysn’ package (Reuman et al. 2022) in the R language, version 3.6.3 (R Core Team 2022).

**TABLE 1:** Summarised results of significant spatial wavelet coherence tests for the variables to which we applied the wavelet Moran theorem. Significance was tested at the *p* < 0.1 threshold (Walter et al. 2020). This table does not include non‐significant coherence results; see Table S2 for full results on the spatial coherence between spread rates and the climate variables in each ecoregion. The *p*‐value and *mean phase* columns come from spatial wavelet coherence tests. The columns *synchrony explained* and *cross term* are an output and diagnostic of the wavelet Moran theorem, respectively. The *Predictor* variables are snow depth (mm; Snow) during the winter and total precipitation (mm; Prcp) during the larval period. *Mean phases* ($\bar{\theta }$), in units of *π* radians, provide information on the phase relationship between the response and predictor variables. The positive sign of the mean phases indicates that synchrony in the response variable preceded that of the predictor variable.

Ecoregion	Timescale	Response	Predictor	*p*	Mean phase ($\bar{\theta }$)	% synchrony explained	Cross term
SUP	2–4	Spread	Prcp	0.0245	0.2419	51.9157	−1.5015
MWP	4–8	Spread	Prcp	0.0484	0.5373	81.3240	9.7784
MWS	2–4	Spread	Snow	0.0565	0.5433	71.0530	−2.0953

**TABLE 2:** Spatial wavelet coherence tests between climate variables and climate indices. These tests revealed whether synchrony in the climate variables (Table 1) was produced by synchrony in multi‐annual climatic fluctuations that occur from teleconnections. The *predictor* variables are the North Atlantic Oscillation (NAO), Pacific Decadal Oscillation (PDO) and the multivariate El Niño Southern Oscillation (MEI) climate indices. The *response* variables are the wintertime climate variable snow depth (mm; Snow) and the larval period variable precipitation (mm; Prcp). Significance was tested at the *p* < 0.1 (Walter et al. 2020). Mean phases ($\bar{\theta }$) in units of *π* radians were provided if meaningful, i.e., when coherence relationships were statistically significant. A positive mean phase value indicates that synchrony in the response variable preceded that of the predictor variable and a negative mean phase, that synchrony in the predictor variable preceded that of the response. All values have been rounded to four digits.

Ecoregion	Predictor	Response	Timescale	*p*	Mean phase ($\bar{\theta }$)
MWS	NAO	Snow	2–4	0.5225	
MWS	PDO	Snow	2–4	0.6783	
MWS	MEI	Snow	2–4	0.0769	−0.9315
SUP	NAO	Prcp	2–4	0.7952	
SUP	PDO	Prcp	2–4	0.0270	0.0125
SUP	MEI	Prcp	2–4	0.9271	

## 3 Results

**FIGURE 2:**
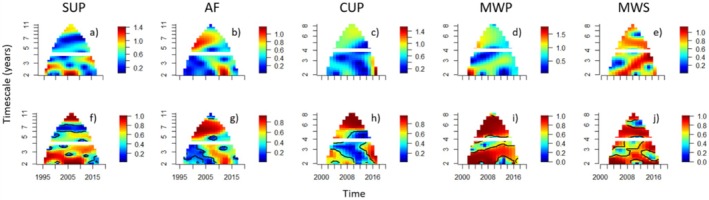
Wavelet mean field (WMF) magnitude (a–e) and wavelet phasor mean field (WPMF) (f–j) plots of the synchrony and phase synchrony (respectively) in rates of spread rate across time and timescale in each ecoregion—the Southeastern USA Plains (SUP), Appalachian Forest (AF), Central USA Plains (CUP), Mixed Wood Plains (MWP) and Mixed Wood Shield (MWS) ecoregions. Values of plots nearest 0 represent weak synchrony and values near 1, stronger synchrony (a–e) or phase synchrony (f–j), at the indicated times and timescales. Mathematically, the time‐averaged WMF magnitude is constrained to the interval (0,1), but it can exceed 1 for some times if it is balanced by times where it is < 1 (Sheppard et al. 2016). The horizontal white line in each plot displays the cutoff between the timescales of interest—short (2–4 years) and long (> 4 years). The black contours (f–j) delineate significant phase synchrony at the *p* < 0.001 threshold (Sheppard et al. 2019). For detailed interpretation about these types of plots please refer to pedagogical figures in Sheppard et al. (2016) (Figure S1) and Anderson et al. (2021) (Figure 1).

Results from the WMFs showed that synchrony in spread was present in all ecoregions but the magnitude, timing and timescale structure of synchrony varied considerably among ecoregions (Figure 2a–e). Across ecoregions, synchrony in spread occurred more episodically at short versus long timescales (Figure 2a–e). The main exception to this was in the two northernmost ecoregions, the MWP (Figure 2d) and the MWS (Figure 2e), where synchrony in spread consistently and similarly shifted from a short timescale towards the 5‐year timescale. At long timescales, the strength of synchrony in spread was generally consistent across the entire time series for an ecoregion. For example, in the southernmost and warmest ecoregion, the SUP, synchrony in spread was generally weak at long timescales (dark blue area of the WMF; Figure 2a). In contrast, in the neighbouring AF, spread displayed strong synchrony across nearly the entire time series at long timescales (red areas of the WMF; Figure 2b). WPMFs confirmed the statistical significance of patterns of synchrony in spread rates for most times and timescales (*p* < 0.001; Figure 2f–j).

**FIGURE 3:**
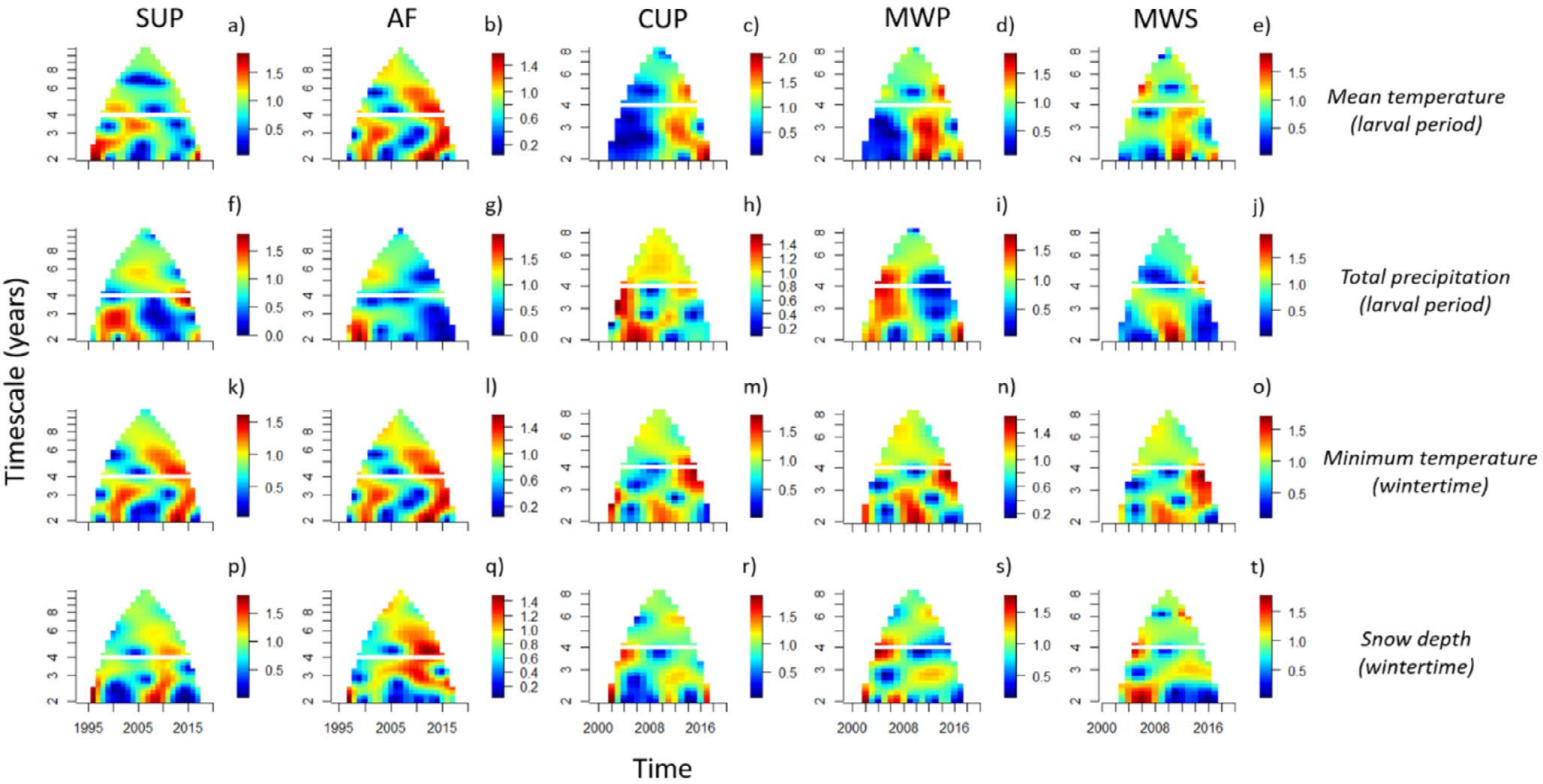
Wavelet mean field (WMF) magnitude plots of time‐ and timescale‐specific spatial synchrony in mean temperature during the larval period (row 1), total precipitation during the larval period (row 2), wintertime minimum temperature (row 3) and wintertime snow depth (row 4). The columns represent the climate variables by ecoregion. The values of the plots that are near 0 represent weak synchrony and values near 1, strong synchrony, at the indicated times and timescales. Mathematically, the time‐averaged WMF magnitude is constrained to the interval (0,1), but it can exceed 1 if it is balanced by times when it is < 1 (Sheppard et al. 2016). The horizontal white line displays the cutoff between the timescales of interest—short (2–4 years) and long (> 4 years). For detailed interpretation about these types of plots please refer to pedagogical figures in Sheppard et al. (2016) (Figure S1) and Anderson et al. (2021) (Figure 1).

Patterns of synchrony for a given climate variable, and their relationships with synchrony in spread rate, tended to be different in northern (i.e., CUP, MWP, MWS) versus southern ecoregions (i.e., AF, SUP) (Figure 3). There were notable similarities between patterns of synchrony for the climate variables (Figure 3) and those found for synchrony in spread rate (Figure 2a–e). In the SUP, for example, patterns of synchrony in spread (Figure 2a) and precipitation (Figure 3f) were similar at short timescales. In the MWS and MWP, patterns of synchrony in spread (Figure 2d,e) were similar to synchrony in snow depth (Figure 3s,t) at both short and long timescales. For most other ecoregions and climate variables, spread and climate generally displayed dissimilar patterns of synchrony.

**FIGURE 4:**
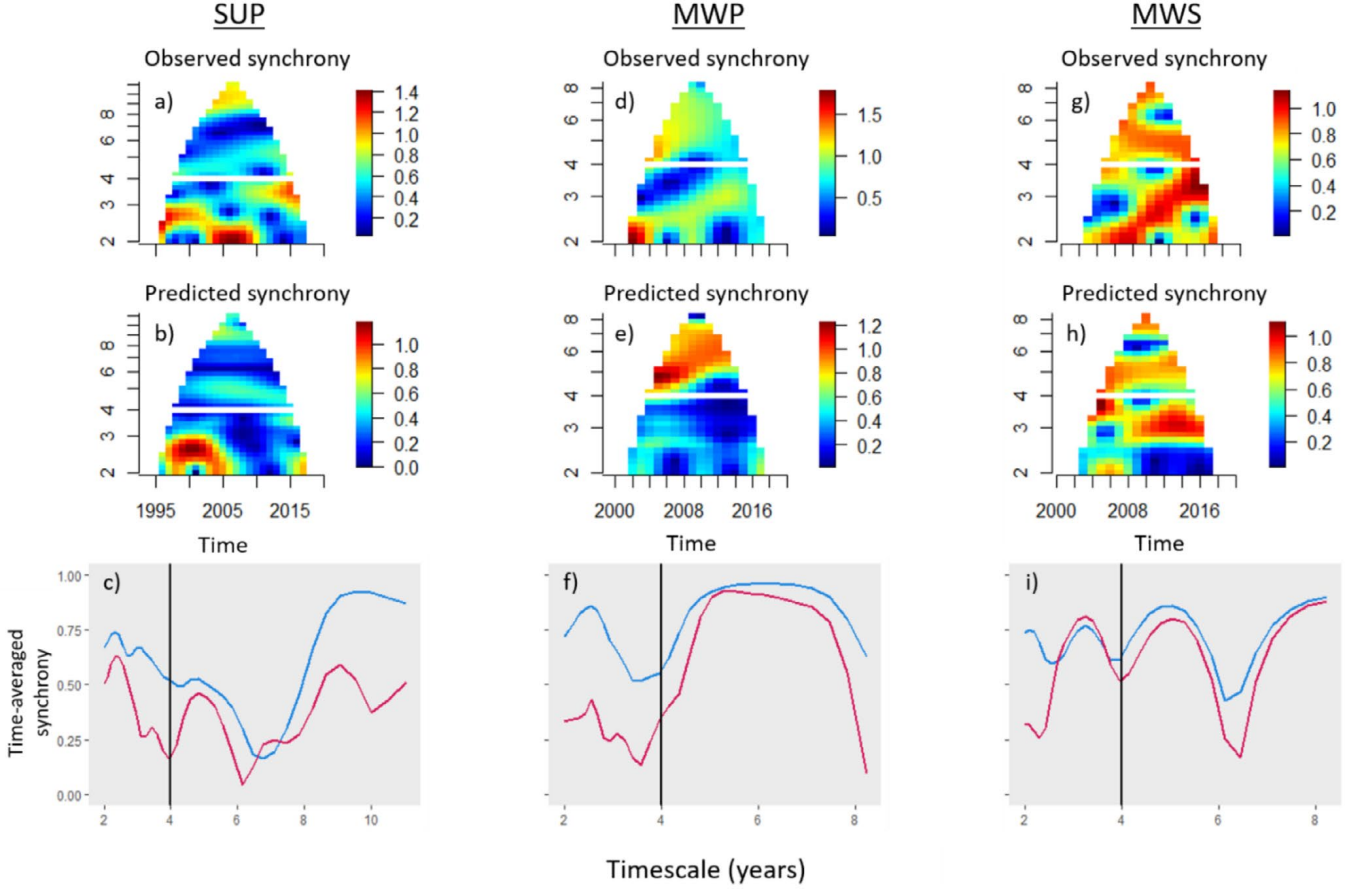
Spatial synchrony in spread is explained by springtime precipitation in the SUP (a–c) and MWP (d–f), and wintertime snow depth in the MWS (g–i). Observed time‐ and timescale‐specific synchrony in spread depicted by wavelet mean fields (WMFs) for the SUP (a), MWP (d), MWS (g). In the SUP and MWP ecoregions precipitation during the laravl period predicted synchrony in spread rate (b, d) (Table 1). In the MWS, snow depth predicted synchrony in spread rate (e) (Table 1). The bottom panels (c, f, i) provide the same information as the WMFs for observed (a, d, g) and predicted (b, e, h) synchrony in spread rate except the timescale‐specific synchrony has been averaged across all years, representing the *mean squared synchrony*. The values of the plots that are near 0 represent weak synchrony and values near 1, strong synchrony, at the indicated times and timescales. Mathematically, the time‐averaged wavelet mean field magnitude is constrained to the interval (0,1), but it can exceed 1 for some times if it is balanced by times where it is < 1 (Sheppard et al. 2016). Horizontal white lines (a, b, d, e, g, h) and black vertical lines (c, f, i) delineate the timescales of interest—short (2–4 years) and long (> 4 years).

Spatial wavelet coherence analysis revealed that only in the two northernmost ecoregions and the southernmost ecoregion was synchrony in spread coherent with any of the climate variables (Table 1 and Table S2). This coherence occurred at short timescales (2–4‐years) in the SUP and MWS, and long timescales (> 4 years) in the MWP. In the SUP, synchrony in spread rate was nearly in‐phase (as indicated by the phase difference, given in units of *π* radians) with precipitation during the larval period at short timescales (*p* = 0.025, $\bar{\theta }$ = 0.242). Specifically, synchronous interannual fluctuations in precipitation explained 52% of synchrony in spread in this ecoregion (Table 1). The synchrony in spread predicted via the wavelet Moran theorem (Figure 4b) was most similar to the observed synchrony at the ≈2–3‐year timescale band (Figure 4a); averaging over time (Figure 4c) underscores the predictive power of this relationship. The multi‐annual synchronous fluctuations in precipitation that drove synchrony in spread were coherent and in‐phase with the PDO (*p* = 0.027, $\bar{\theta }$ = 0.0125; Table 2).

In the MWP ecoregion, synchrony in precipitation during the larval period and synchrony in spread rate were coherent with each other at long timescales (*p* = 0.048), with synchrony in precipitation explaining 81% of synchrony in spread (Table 1). The mean phase difference indicated an approximate quarter‐cycle phase shift ($\bar{\theta }$ = 0.537) between spread rate and precipitation. This means that spread rate declined a quarter‐cycle (~1 year) after years with increasing precipitation. Precipitation best predicted synchrony in spread at ≈5–7‐year timescales (Figure 4d,e). There was no relationship between synchrony in precipitation and any of the teleconnection indices.

Just north of the MWP, in the MWS, synchrony in winter snow depth explained 71% of synchrony in spread rate at short timescales (Table 1). These two variables were coherent with each other at the *p* < 0.1 significance threshold (*p* = 0.057). This relationship displayed an approximate quarter‐cycle phase shift ($\bar{\theta }$ = 0.543); spread rate declined ~1 year following years with increasing snow depth. The relationship between synchrony in spread and synchrony in snow depth had greatest predictive power at ≈3–4‐year timescales (Figure 4f–h). Multi‐annual synchronous fluctuations in snow depth were in an anti‐phase (negative) relationship with the MEI climate index (*p* = 0.077, $\bar{\theta }$ = −0.9315; Table 2).

## 4 Discussion

By leveraging unparalleled data on low‐density populations along a species' range edge, we provide evidence of spatial synchrony in rates of invasive spread by the spongy moth, a major forest pest in North America. Although synchrony in population growth rates and abundance is ubiquitous across many taxa (Bjørnstad et al. 1999; Hanski and Woiwod 1993; Hudson and Cattadori 1999; Liebhold et al. 2004; Ranta et al. 1995), and population growth is an important component of spread (Skellam 1951), to our knowledge this is the first study to demonstrate the phenomenon of spatial synchrony for rates of spread. We revealed that multi‐annual synchronised fluctuations in climate explained considerable portions of spatial synchrony in rates of range expansion and contraction in three of five ecoregions (Table 1). Further, the climate variables that explained synchrony in spread in the SUP and MWS were significantly coherent with teleconnection patterns (Table 2). These findings align with past studies showing that teleconnection patterns produce multi‐annual, synchronised fluctuations in local climate conditions that drive population synchrony at similar timescales (Anderson et al. 2021; Castorani et al. 2022; Sheppard et al. 2016) and provide evidence that teleconnections influence spatiotemporal patterns of invasive spread.

Synchrony in spread could only be attributed to a tested climate variable in the MWS, MWP, and SUP ecoregions (Table 1). In the MWS, there was a phase‐lagged negative relationship between snow depth and spread, with peaks in spread ≈1 year after periods of low snow depth. This result is inconsistent with our prediction that spread rate would increase with increasing snow depth, based on past studies that suggested snow cover may insulate overwintering eggs against lethal temperatures during the winter, thereby increasing their survival (Madrid and Stewart 1981; Streifel et al. 2019). Higher snow depth may have been associated with adverse conditions such as below‐average winter temperatures or freezing temperatures after the emergence of larvae in the spring, possibly outweighing the positive effects of snow for survival. Supporting this explanation is our finding that increased snow depth in the MWS was associated with lower MEI index values (Table 2), La Niña conditions, which are associated with below‐average winter temperatures in the northern United States (Yu et al. 2012). In the MWP ecoregion, south of the MWS, timescale‐specific patterns of synchrony in spread (Figure 2d) and snow depth (Figure 3s) were very similar to those in the MWS (Figures 2e and 3t), although there were no statistically significant effects of snow depth on spread rates (Table S2). Together, these findings show that snow depth is an important predictor of spread along the northernmost range limits of the spongy moth.

In the MWP, periods of low spread rate lagged roughly 1 year behind years with increased precipitation, which aligns with our prediction of a negative effect of precipitation on spread rate due to higher larval mortality from the fungal‐pathogen *E. maimaiga* when environmental moisture is high (Hajek 1999). Although larval infections by *E. maimaiga* have occurred throughout much of the spongy moth's invasive range (Hajek et al. 2021), parts of the range, especially nearest the range limits, may be either too cold or too warm for widespread persistence of the fungus (Rodenberg et al. 2024). This may partially explain how effects of precipitation on spongy moth spread varied among ecoregions.

In the SUP, synchronous peaks in spread roughly coincided with synchronous peaks in precipitation, possibly due to effects of weather on dispersal. Increased precipitation may be associated with storm events that cause strong winds to transport individuals from higher‐density established populations towards the invasion front (Tobin and Blackburn 2008). Frank et al. (2013) concluded that historically high rates of spongy moth spread in Wisconsin occurred because strong winds during storms blew early‐instar larvae across Lake Michigan from high‐density populations that originated in Michigan. Similarly, it is believed that wind‐aided, long‐distance dispersal has facilitated invasive spread by two *Drosophila* species, *D. suzukii* (Asplen et al. 2015) and *D. melanogaster* (Leitch et al. 2021). In the SUP, we found a marked positive relationship between multi‐annual synchronised fluctuations in precipitation and the PDO (Table 2); ‘warm’ phases of the PDO (positive PDO values) are associated with increased precipitation in the southeastern United States (Wei et al. 2021). The PDO has typically exhibited decadal to multi‐decadal variability, but recent research suggests a shift in these long timescale cycles towards shorter ones beginning in 1999 (Li et al. 2020; Zhang and Delworth 2015, 2016). The PDO has been in a strong warm phase since 2014, but between 1999 and 2014, PDO phases were roughly 2–4 years long, well within the range of timescales examined in this study.

Significant relationships between synchrony in spread and climate were only found in the three ecoregions with the highest annual variabilities in spread rates (km yr^−1^), the MWS (SD = 37.2 km yr^−1^), SUP (SD = 39.1 km yr^−1^), and MWP (SD = 22.8 km yr^−1^), with no such relationships found in the AF (SD = 20.9 km yr^−1^) or CUP (SD = 18.7 km yr^−1^). Across the ecoregions, there were no marked differences in the amount of annual variability for the climate variables (Table S3). Greater variability in rates of spread in the two northernmost ecoregions (MWS and MWP) and the southernmost ecoregion (SUP) may be due to higher variability in population growth rates from more frequent exposure to extreme weather conditions at the northern and southern limits of the range. In aphid and moth species, Hanski (1990) found, and Hanski and Woiwod (1993) later confirmed, a positive relationship between annual variability in population abundances and spatial population synchrony. This relationship suggested that regionally correlated environmental stochasticity simultaneously increased both spatial population synchrony and population variability (Hanski and Woiwod 1993). Given that population growth is a critical component of invasive spread (Skellam 1951), the strongest effects of synchrony in weather on rates of spread may occur where weather exhibits its strongest effects on growth rates.

This study demonstrates spatial synchrony in range expansion. Just as climatic drivers can produce synchrony in population growth or abundance, we show that synchronised fluctuations in seasonal climate conditions at multi‐annual timescales can synchronise invasive spread. In addition, at the northern and southern extremes of the species' range, the synchronising effects of climate on spread were linked to teleconnection patterns. A recent review by Wan et al. (2022) highlighted the importance of using information on the phase and strength of teleconnection indices to anticipate changes in spatial population synchrony driven by multi‐annual climatic patterns. Although predictability in the phase and strength of teleconnections is generally limited to seasonal timescales (Williams et al. 2023), managers focused on slowing the spread of the spongy moth could use seasonal projections of climate indices to anticipate when synchronised fluctuations in spread will occur, especially near the latitudinal extremes of the range front. It is unknown whether synchrony in spread is a widespread phenomenon and if similar relationships between teleconnections and synchrony in spread exist for other species. If prevalent, this phenomenon could prove critical to management efforts aimed at either controlling the spread of pests and disease vectors or facilitating the movement of rare species as the climate changes.

## Author Contributions

C.A.R. and K.J.H. conceived the idea. All authors contributed to development of the analytical methods. C.A.R. performed the data assimilation, analyses and wrote the first draft of the manuscript. All authors contributed to review and editing of the manuscript.

## Supporting information


Data S1.

## Data Availability

All data and codes supporting the results of this manuscript are archived in the Dryad public repository https://doi.org/10.5061/dryad.v6wwpzh56. All the computer code behind the analyses and results of this paper are archived at https://github.com/clare‐a‐rodenberg/synchrony‐spread‐climate.git.
