# Supplementary material for: Evidence of Spatial Synchrony in the Spread of an Invasive Forest Pest
Source: Ecol Lett. 2025 May 30;28(6):e70140. doi: 10.1111/ele.70140 (PMC12123481; doi:10.1111/ele.70140)
Supplement: Supplementary file 1 — Data S1. [file ELE-28-0-s001.pdf]

Supplemental tables and figures for manuscript: **Evidence of spatial synchrony in the spread of an invasive forest pest: Supporting information** – *Rodenberg, Walter, Haynes*

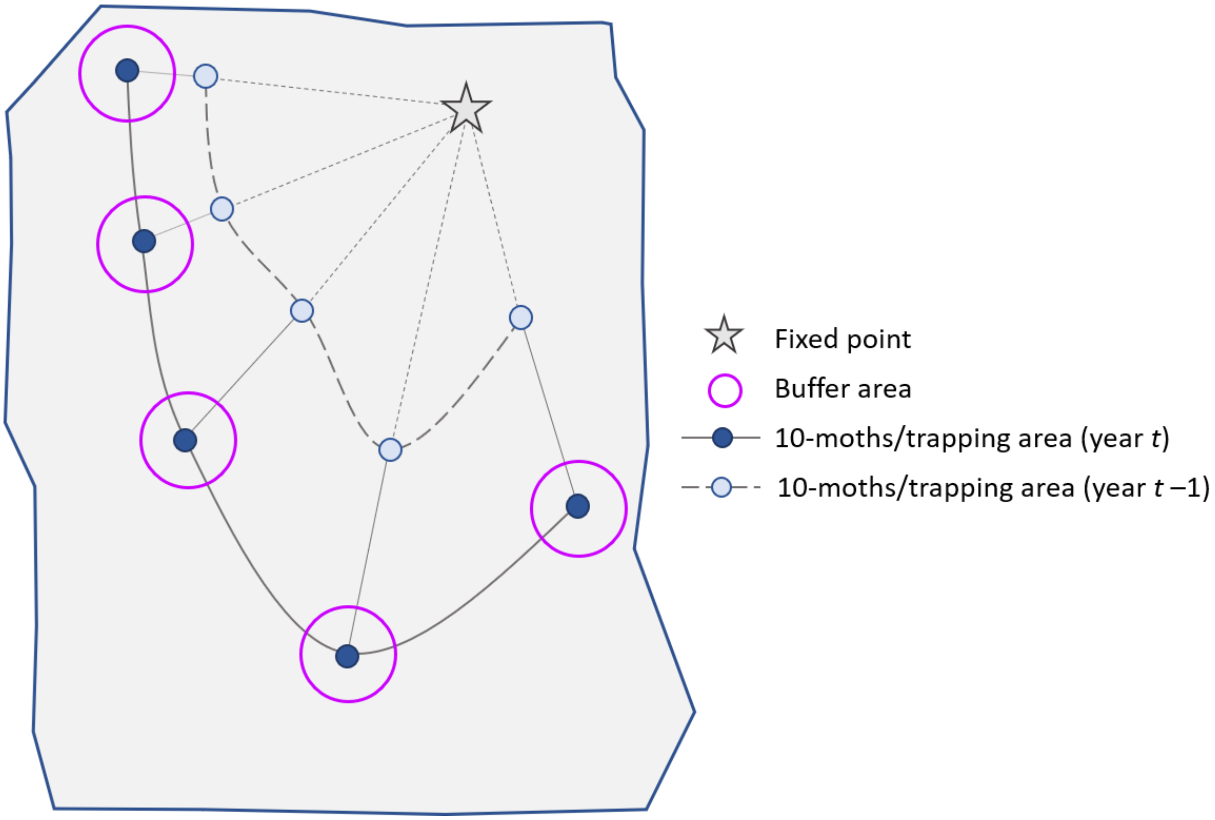

Figure S1: Conceptual diagram that illustrates 1) how rate of spread was estimated and 2) the areas from which data on the explanatory climate variables (temperature, precipitation, snow depth) were extracted and spatially attributed to the spread rate data. Rate of spread was estimated based on the displacement between 10-moth lines in year t – 1 and year t (boundary displacement method), for each transect radiating from the fixed point. The mean annual value of each explanatory variable within a buffer centered at the intersection of each transect and the 10-moth line was calculated to examine the effects of synchrony in climate on synchrony in invasive spread. The buffer size was equal to the average rate of spread that occurred in an ecoregion for each study period (see Table 1 for information on the study periods).

Table S1: Total number of transects for which there were consistent data through study period for each ecoregion.

Ecoregion	Transects	Years
Southeastern USA Plains (SUP)	47	1993-2020
Appalachian Forest (AF)	85	1994-2020
Central USA Plains (CUP)	25	1999-2020
Mixed Wood Plains (MWP)	10	1999-2020
Mixed Wood Shield (MWS)	10	2000-2020

Table S2: Results from spatial wavelet coherence tests on all pairwise combinations of spread rate and each abiotic variable and defoliation. The *P-value* and *mean phase* columns are test statistics from the coherence tests. To identify potential drivers of synchrony in spread rates, coherences tests were performed on the relationship between synchrony in spread rate (response variable) and synchrony in each climate variable (predictor variables). The *Predictor* variables are the wintertime climate variables minimum temperature (°C; Tmin) and snow depth (mm; Snow) and the larval period variables mean temperature (°C; Tmean) and total precipitation (mm; Prcp). Significance was tested at the $P < 0.1$ in accordance with Walter et al. 2020. Mean phases ($\bar{\theta}$) in units of π radians were only provided if meaningful, i.e., when coherence relationships were significant. The positive sign of the mean phases indicates that synchrony in the response variable preceded that of the predictor variable. All values have been rounded to four digits.

Ecoregion	Response	Predictor	Timescale	*P*-value	Mean phase ($\bar{\theta}$)
MWS	Spread	Tmin	2-4	0.3872	
MWS	Spread	Tmin	4-8	0.3559	
MWS	Spread	Tmean	2-4	0.3752	
MWS	Spread	Tmean	4-8	0.9334	
MWS	Spread	Prcp	2-4	0.3079	
MWS	Spread	Prcp	4-8	0.7459	
MWS	Spread	Snow	2-4	0.0565	0.5433
MWS	Spread	Snow	4-8	0.4528	
MWP	Spread	Tmin	2-4	0.8298	
MWP	Spread	Tmin	4-8	0.1962	
MWP	Spread	Tmean	2-4	0.5512	
MWP	Spread	Tmean	4-8	0.5487	
MWP	Spread	Prcp	2-4	0.8721	
MWP	Spread	Prcp	4-8	0.0485	0.5373
MWP	Spread	Snow	2-4	0.1134	
MWP	Spread	Snow	4-8	0.2084	
CUP	Spread	Tmin	2-4	0.7379	
CUP	Spread	Tmin	4-8	0.2909	
CUP	Spread	Tmean	2-4	0.9619	
CUP	Spread	Tmean	4-8	0.5552	

CUP	Spread	Prcp	2-4	0.9105	
CUP	Spread	Prcp	4-8	0.2659	
CUP	Spread	Snow	2-4	0.9916	
CUP	Spread	Snow	4-8	0.3103	
AF	Spread	Tmin	2-4	0.2048	
AF	Spread	Tmin	4-11	0.4386	
AF	Spread	Tmean	2-4	0.8172	
AF	Spread	Tmean	4-11	0.3976	
AF	Spread	Prcp	2-4	0.9760	
AF	Spread	Prcp	4-11	0.2448	
AF	Spread	Snow	2-4	0.3606	
AF	Spread	Snow	4-11	0.2707	
SUP	Spread	Tmin	2-4	0.9350	
SUP	Spread	Tmin	4-11	0.8456	
SUP	Spread	Tmean	2-4	0.3028	
SUP	Spread	Tmean	4-11	0.9165	
SUP	Spread	Prcp	2-4	0.0245	0.2419
SUP	Spread	Prcp	4-11	0.9465	
SUP	Spread	Snow	2-4	0.7676	
SUP	Spread	Snow	4-11	0.7031	

Table S3: Annual variability (±SD) in spread rates (km yr^{-1}) for each of the ecoregions (*Spread* column) compared to annual variability in the climate variables tested. Climate variables are represented by the columns *Tmean* (mean temperature during the larval period; °C), *Prcp* (precipitation during the larval period; mm), *Tmin* (minimum wintertime temperature; °C), and *Snow* (snow depth; mm).

Ecoregion	Spread	Tmean	Prcp	Tmin	Snow
SUP	41.3	1.1	90	1.4	1504.1
AF	20.9	1.4	101.2	1.7	4170
CUP	18.7	0.9	104.8	2.16	5135.9
MWP	22.8	1.2	63.1	2.1	7021.1
MWS	37.2	1.1	71	2.16	6139.6

References

1. Tobin, P. C., & Blackburn, L. M. (2014). Long-distance dispersal of the gypsy moth (Lepidoptera: Lymantriidae) facilitated its initial invasion of Wisconsin. *Environmental Entomology*, 37, 87–93.
2. Walter, J. A., Sheppard, L. W., Venugopal, P. D., Reuman, D. C., Dively, G., Tooker, J. F., *et al.* (2020). Weather and regional crop composition variation drive spatial synchrony of lepidopteran agricultural pests. *Ecological Entomology*, 45, 573–582.
